# SPSS Syntax for Combining Results of Principal Component Analysis of Multiply Imputed Data Sets using Generalized Procrustes Analysis

**DOI:** 10.1177/0146621621990757

**Published:** 2021-02-04

**Authors:** Bart van Wingerde, Joost van Ginkel

**Affiliations:** 1 Leiden University, The Netherlands

**Keywords:** missing data, multiple imputation, principal component analysis, generalized procrustes analysis

## Description

Multiple imputation (Rubin, 1987) is a well-known method for handling missing data. Applying the procedure to an incomplete data set results in several plausible complete versions of the incomplete data set which are then all analyzed with the same statistical analysis. In order to obtain one overall analysis that is used for interpretation, the analysis results of these several completed data sets are combined using specific combination procedures. For principal component analysis (PCA), Van Ginkel and Kroonenberg (2014) proposed generalized procrustes analysis (GPA; Gower, 1975; Ten Berge, 1977) to combine the results. To date, GPA seems to have been little used for combining PCA results in multiply imputed data sets, as shown from relatively few citations of Van Ginkel and Kroonenberg (2014) by applied research papers. One reason could be that there are only few software packages that have implemented GPA. Exceptions are the “shapes” package (Dryden & Mardia, 2016) in R (R Core Team, 2018) and the stand-alone program 3WayPack (Kroonenberg & De Roo, 2010). In addition, these software packages may not be well known by applied researchers, and it may not be obvious to them that they may also be used for combining the results of PCA in multiply imputed data. For these researchers, the authors developed a user-friendly SPSS subroutine which is specifically aimed at combining the results of PCA, as described by Van Ginkel and Kroonenberg (2014), and which can be applied completely within SPSS. To run the subroutine, one must first carry out a PCA on each of the imputed data sets in SPSS and save the results to a data file. Next, the subroutine may carry out the combining of the saved results using GPA. Within the subroutine, a number of required arguments and some optional arguments are specified. Among the most important optional arguments are the display of the Varimax rotated centroid solution in the output, and the display of loading plots of both the unrotated and Varimax rotated centroid solutions, along with their convex hulls (Van Ginkel & Kroonenberg, 2014).

## Availability

The SPSS syntax file “GPA.sps” contains the subroutine that was partly written in SPSS for Windows using the MATRIX command (SPSS inc, 2017) and partly in Python. The routine is included in a zip file called GPA.zip, available free of charge from https://www.universiteitleiden.nl/en/staffmembers/joost-van-ginkel#tab-1. A manual of the subroutine is also included in this zip file. The syntax files can be applied using SPSS 22.0 and later versions for Windows. SPSS 21.0 has the option to install SPSS Python Essentials during setup. For SPSS versions 18.0, 19.0, and 20.0, SPSS Python Essentials can be downloaded from the IBM SPSS website.

## Supplemental Material

sj-pdf-3-apm-10.1177_0146621621990757 – Supplemental material for SPSS Syntax for Combining Results of Principal Component Analysis of Multiply Imputed Data Sets using Generalized Procrustes Analysis Click here for additional data file. Supplemental material, sj-pdf-3-apm-10.1177_0146621621990757 for SPSS Syntax for Combining Results of Principal Component Analysis of Multiply Imputed Data Sets using Generalized Procrustes Analysis by Bart van Wingerde and Joost van Ginkel in Applied Psychological Measurement

sj-sav-1-apm-10.1177_0146621621990757 – Supplemental material for SPSS Syntax for Combining Results of Principal Component Analysis of Multiply Imputed Data Sets using Generalized Procrustes Analysis Click here for additional data file. Supplemental material, sj-sav-1-apm-10.1177_0146621621990757 for SPSS Syntax for Combining Results of Principal Component Analysis of Multiply Imputed Data Sets using Generalized Procrustes Analysis by Bart van Wingerde and Joost van Ginkel in Applied Psychological Measurement

sj-sav-4-apm-10.1177_0146621621990757 – Supplemental material for SPSS Syntax for Combining Results of Principal Component Analysis of Multiply Imputed Data Sets using Generalized Procrustes Analysis Click here for additional data file. Supplemental material, sj-sav-4-apm-10.1177_0146621621990757 for SPSS Syntax for Combining Results of Principal Component Analysis of Multiply Imputed Data Sets using Generalized Procrustes Analysis by Bart van Wingerde and Joost van Ginkel in Applied Psychological Measurement

sj-sps-2-apm-10.1177_0146621621990757 – Supplemental material for SPSS Syntax for Combining Results of Principal Component Analysis of Multiply Imputed Data Sets using Generalized Procrustes Analysis Click here for additional data file. Supplemental material, sj-sps-2-apm-10.1177_0146621621990757 for SPSS Syntax for Combining Results of Principal Component Analysis of Multiply Imputed Data Sets using Generalized Procrustes Analysis by Bart van Wingerde and Joost van Ginkel in Applied Psychological Measurement

sj-sps-5-apm-10.1177_0146621621990757 – Supplemental material for SPSS Syntax for Combining Results of Principal Component Analysis of Multiply Imputed Data Sets using Generalized Procrustes Analysis Click here for additional data file. Supplemental material, sj-sps-5-apm-10.1177_0146621621990757 for SPSS Syntax for Combining Results of Principal Component Analysis of Multiply Imputed Data Sets using Generalized Procrustes Analysis by Bart van Wingerde and Joost van Ginkel in Applied Psychological Measurement
