# Supplementary material for: SPSS Syntax for Combining Results of Principal Component Analysis of Multiply Imputed Data Sets using Generalized Procrustes Analysis
Source: Appl Psychol Meas. 2021 Feb 4;45(3):231–2. doi: 10.1177/0146621621990757 (PMC8042555; doi:10.1177/0146621621990757)
Supplement: sj-pdf-3-apm-10.1177_0146621621990757 – Supplemental material for SPSS Syntax for Combining Results of Principal Component Analysis of Multiply Imputed Data Sets using Generalized Procrustes Analysis [file sj-pdf-3-apm-10.1177_0146621621990757.pdf]

SPSS Syntax for Combining Principal Component Loadings in Multiple Imputation using Generalized Procrustes Analysis

Authors: To be revealed after acceptance

October 20, 2020

1 Contents of the zip file

This zip file contains the following files:

- `GPAmanual.pdf`: this file
- `Run_GPA.sps`: SPSS syntax file. This file can be modified to suit your needs.
- `GPA.sps`: SPSS syntax file. The file which performs all the calculations. This is a read only file.
- `incomplete2_imp.sav`: A multiple-imputation data set in SPSS format containing the responses of 300 'respondents' to 41 items, denoted `V1`, ..., `V41`. The original incomplete data set is a simulated data set from a simulation study by Van Ginkel, Van der Ark & Sijtsma (2007). The data file contains the original incomplete data set, plus five completed versions of the incomplete data set. The different versions are indicated by an additional variable `imputation_`, which contains the data set number (0 indicating the original incomplete data set). The five completed versions were created using multiple Two-Way imputation for separate scales (Van Ginkel, Van der Ark, & Sijtsma, 2007). Variables `V1` to `V40` have ordered answer categories ranging from 0 to 4, variable `V41` is a dichotomous variable with values 1 and 2. Five percent of the scores are missing. Missing values are indicated by a comma.

- `ExamplePCA.sav`: An example SPSS data file containing the results of five principal component analyses resulting from five completed data sets. .

2 About the SPSS Syntax

2.1 The Purpose

The purpose of this SPSS syntax is to combine the results of principal component analysis (PCA) on a multiply imputed dataset using Generalized Procrustes Analysis (GPA; Gower, 1975; Ten Berge, 1977; Van Ginkel & Kroonenberg, 2014). Rubin (1987) defined several rules for combining the results of multiply imputed datasets. When it comes to combining parameter estimates into one overall estimate, these rules come down to averaging the parameter estimates across the imputed datasets. However, averaging the component loadings in a PCA may lead to several problems. The first problem is that the order of the components may not be the same across imputed datasets (Van Ginkel & Kroonenberg, 2014). A second problem is that when a test contains about the same number of indicative as counterindicative items, in some imputed dataset the signs of the loadings may have been flipped compared to the other imputed datasets (Van Ginkel & Kroonenberg, 2014). The third problem is that if a rotation (e.g. the Varimax rotation) is used, the rotation will be optimized for that specific imputed dataset. However, the mean of those solutions will not be the same as the solution you would get if the components were rotated after combining the imputed datasets. Using GPA solves all three of these problems. GPA will produce a centroid of the different PCA outcomes which acts as an optimal average solution. This centroid solution can then be rotated, for example, by using a Varimax rotation.

Besides providing an overall PCA solution for multiply imputed datasets, this SPSS syntax also gives the option of showing the spread of the component loadings across the imputed datasets. The SPSS syntax does this by showing the area in which the component loadings of all the imputed datasets fall. These areas are called convex hulls. The area of these convex hulls is indicative of the uncertainty caused by the missing data in the dataset (Van Ginkel & Kroonenberg, 2014).

2.2 Generalized Procrustes Analysis

In GPA a transformation matrix for each imputed dataset is computed, which minimizes the sum of the squared distances between the transformed loadings of the imputed data. Let $\mathbf{A}_m$ be a matrix containing the PCA loadings of the m'th imputed dataset ($m = 1, ..., M$), and $\mathbf{T}_m$ the transformation matrix for the m'th imputed dataset. The transformation matrices are computed by minimizing the following function, using an algorithm by Ten Berge (1977).

$$f(\mathbf{T}_1, ..., \mathbf{T}_M) = \sum_{i<j} tr(\mathbf{A}_i\mathbf{T}_i - \mathbf{A}_j\mathbf{T}_j)'(\mathbf{A}_i\mathbf{T}_i - \mathbf{A}_j\mathbf{T}_j). \quad (1)$$

The mean of the M transformed loadings is called the centroid solution. The centroid solution is used as the combined solution for the imputed datasets.

For more information on the algorithm for Generalized Procrustes Analysis, see Gower (1975), Ten Berge (1977), and Van Ginkel and Kroonenberg (2014).

2.3 Disclaimer and Bugs

It should be emphasized that this SPSS syntax is distributed without any warranty on the part of the authors. Although the SPSS syntax has been tested thoroughly, one can never fully exclude the possibility of errors. The authors appreciate suggestions and reports of detected errors (please enclose SPSS data file). All correspondence can be sent to

Address to be revealed after acceptance

3 Using the SPSS Syntax

3.1 Requirements

This SPSS syntax uses the programming language Python. Python is included in the standard installation of SPSS version 22.0 and later versions. SPSS 21.0 has the option to install SPSS Python Essentials during setup. For SPSS versions 18.0, 19.0 and 20.0 SPSS Python Essentials can be downloaded from the IBM SPSS website. The site spss-tutorials.com has links to Python plugins for SPSS versions 15.0 and 17.0.

Figure 1: *Selecting the* `Split File` *Option.*

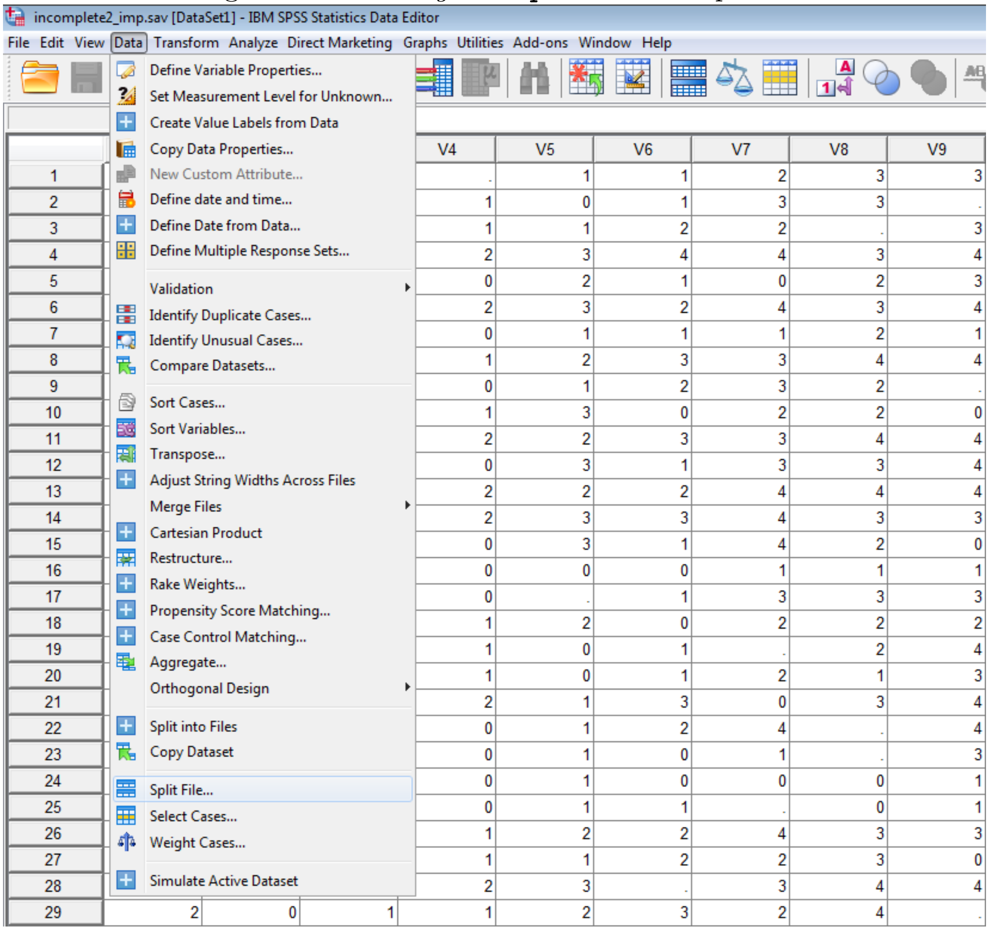

3.2 Preparing your SPSS File

Several steps must be taken before the component loadings can be combined using the SPSS syntax file. After imputing the data, a PCA needs to be carried out on each imputed dataset separately. Next, the output of these PCAs must be saved to an SPSS data file. These steps are explained in the following sections and illustrated using the file `Incomplete2_imp.sav`.

3.2.1 Split File

`Incomplete2_imp.sav` is a file containing multiple imputed versions of the incomplete dataset. The PCA needs to be carried out on each imputed dataset separately. To do this, we must split the dataset by the imputation number in SPSS. Select the `Split File` option in the `Data` task bar. Select

Figure 2: *Choosing* `imputation_` *as Grouping Variable.*

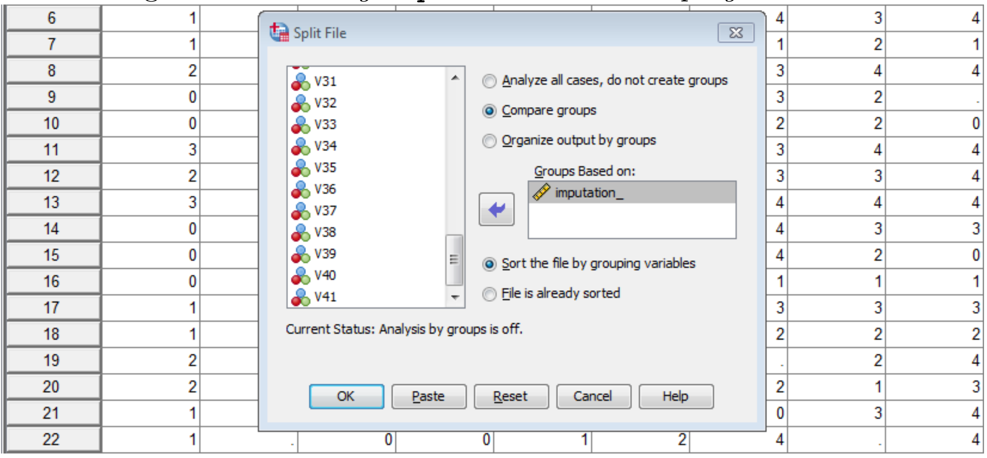

Figure 3: *Selecting the* `OMS` *Option.*

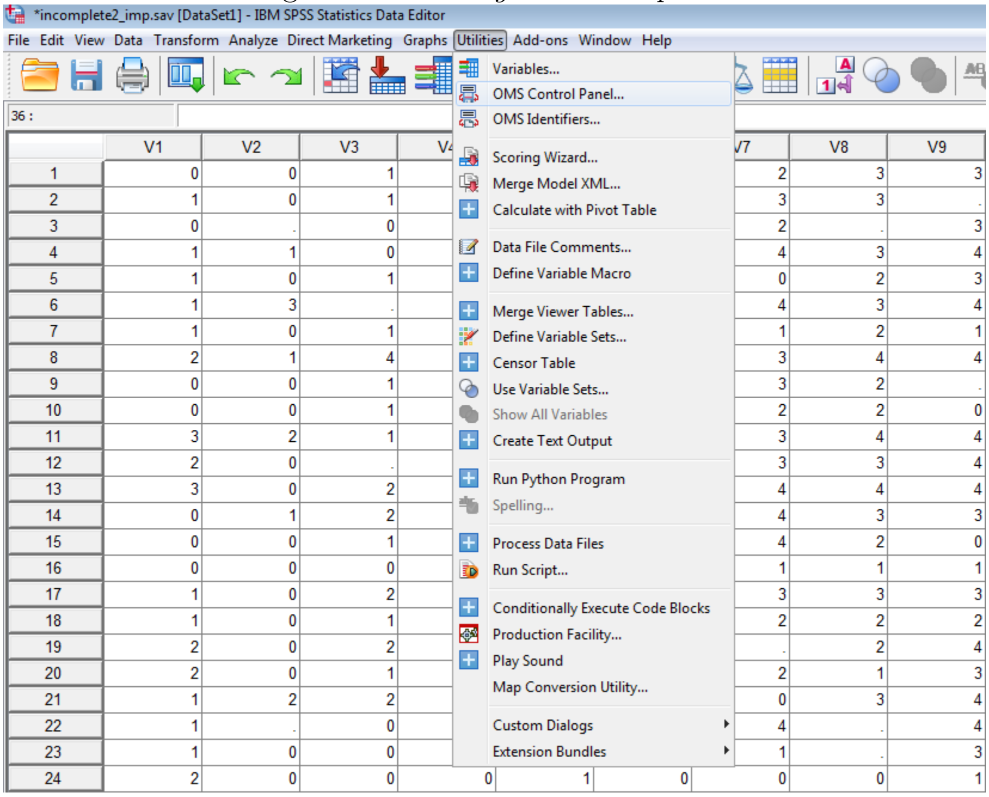

`Compare groups` and add `imputation_` as the grouping variable.

Figure 4: *Selecting Options within* OMS.

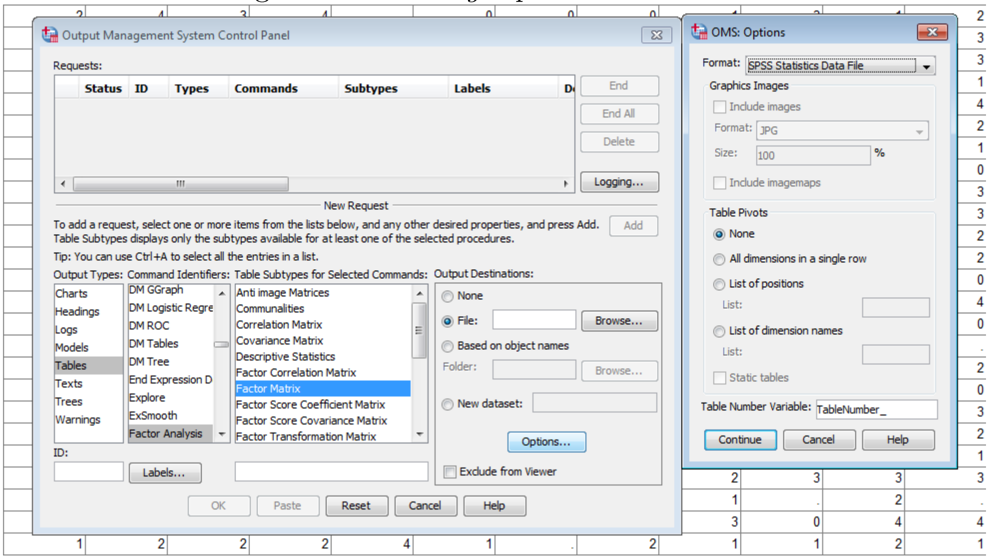

Figure 5: *Confirming the start of* OMS.

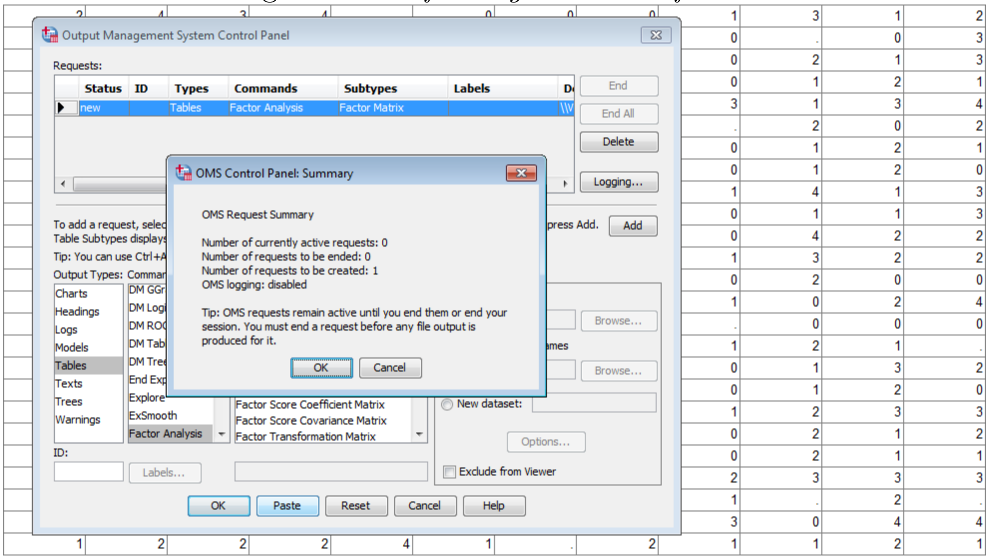

These steps are shown in Figure 1 and Figure 2. Alternatively, one can use the following syntax command:

```
SORT CASES BY imputation_.
SPLIT FILE LAYERED BY imputation_.
```

Figure 6: *Selecting Factor Analysis.*

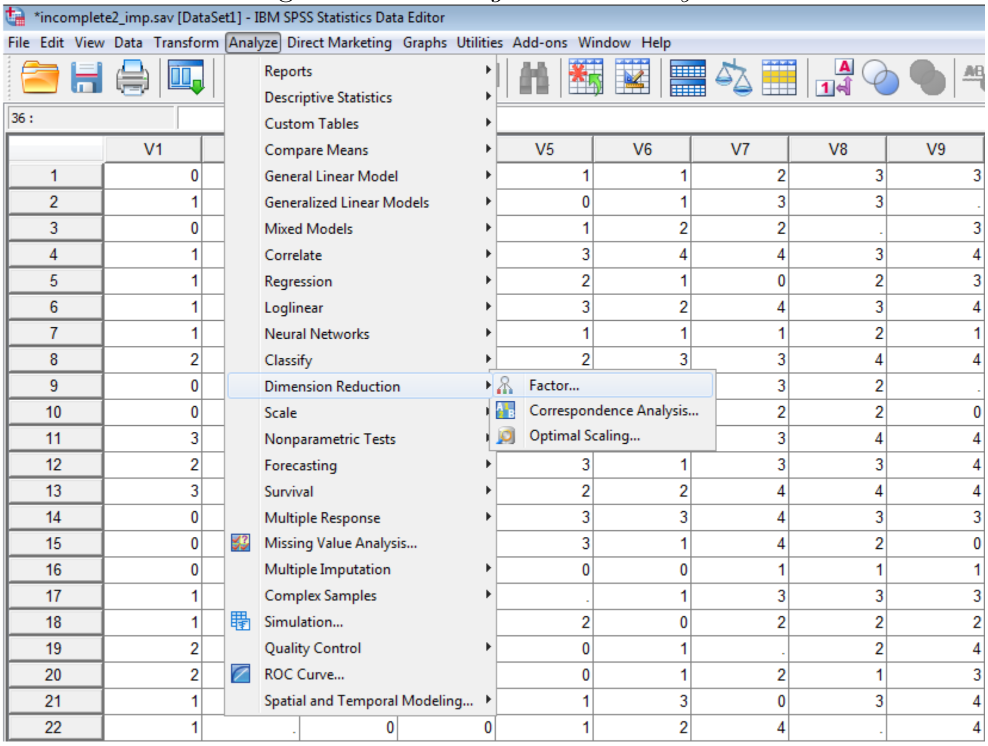

3.2.2 OMS

The `OMS` (Output Management System) option allows us to save the output of the analyses to an SPSS data file. To use this option, select `OMS Control Panel` from the `Utilities` task bar (Figure 3). Next, select `Tables`, `Factor Analysis` and `Factor Matrix`.

Under `Output Destinations` select `File` and specify a file name and location of your choosing. Next, click on `Options` and select `SPSS Statistics Data File` as format. These steps are shown in Figure 4. Lastly, click on `Add` and close the window by clicking `OK` twice (Figure 5).

Next, carry out the principal component analysis for the multiply imputed dataset. This can be done by means of `Analyze`, `Dimension Reduction`, `Factor` (Figure 6). Add `V1` to `V40` to the `Variables` box. Click on `Extraction` and set factors to `Fixed number of factors to extract` to the number of components you want to extract (Figure 7).

Do not base the numbers of components on the Eigenvalue criterion, as different imputations can lead to a different number of extracted factors with the Eigenvalue method. Four components were used in our example. Next

Figure 7: *Selecting the Factor Analysis Options*

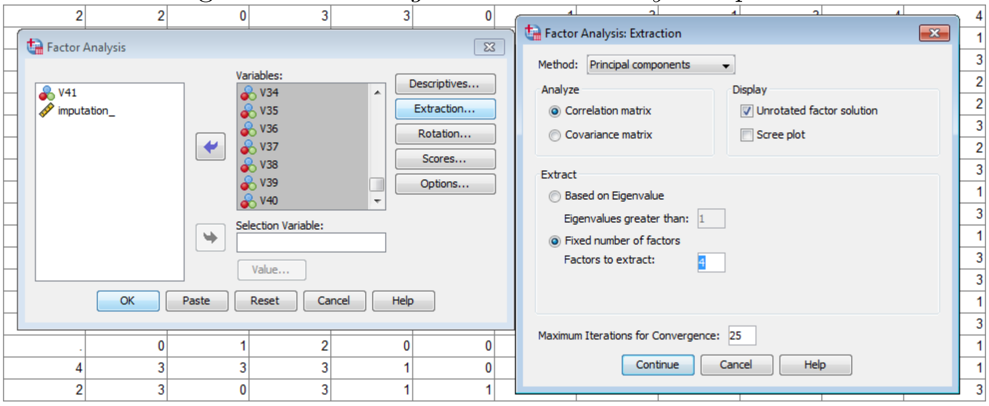

Figure 8: *Ending* `OMS`.

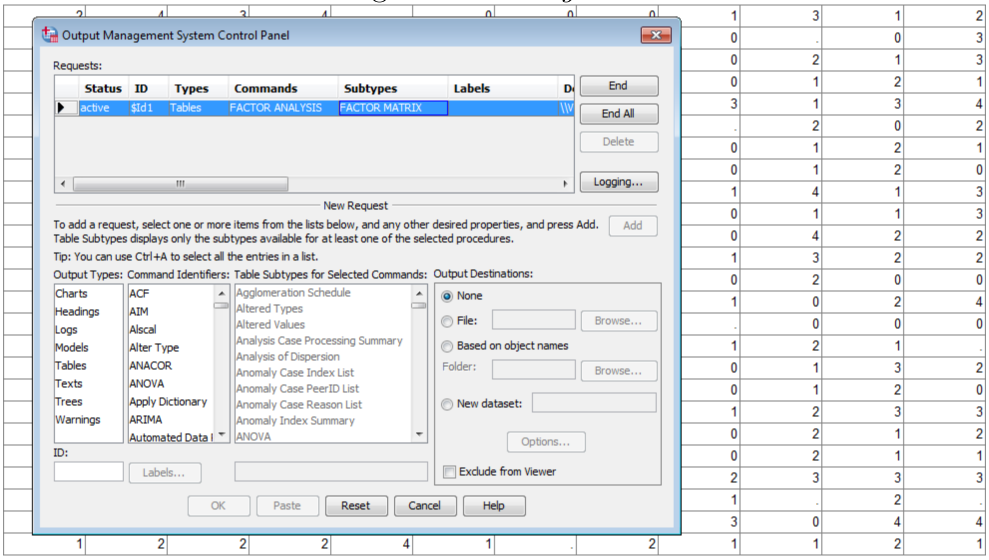

click on `OK`. The last step is to turn off `OMS` logging by going back to the `OMS Control Panel` under utilities and clicking on end (Figure 8). Close this window by clicking `OK` twice (Figure 9).

If the steps are followed correctly on the `Incomplete2_imp.sav` file, the newly created file will look like `ExamplePCA.sav` (Figure 10).

Figure 9: *Confirming the End of `OMS`.*

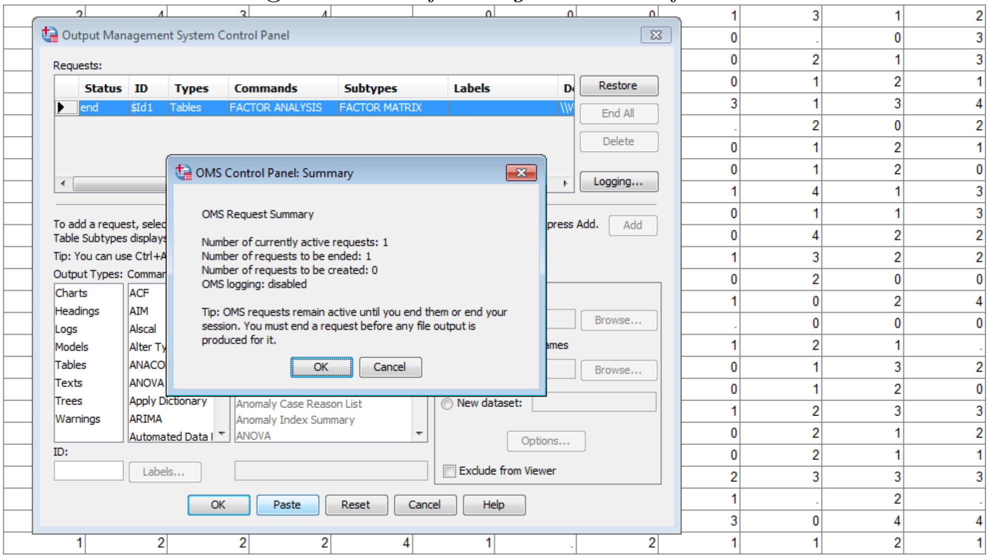

Figure 10: *Example of a file with factor matrices created with the `OMS` Option.*

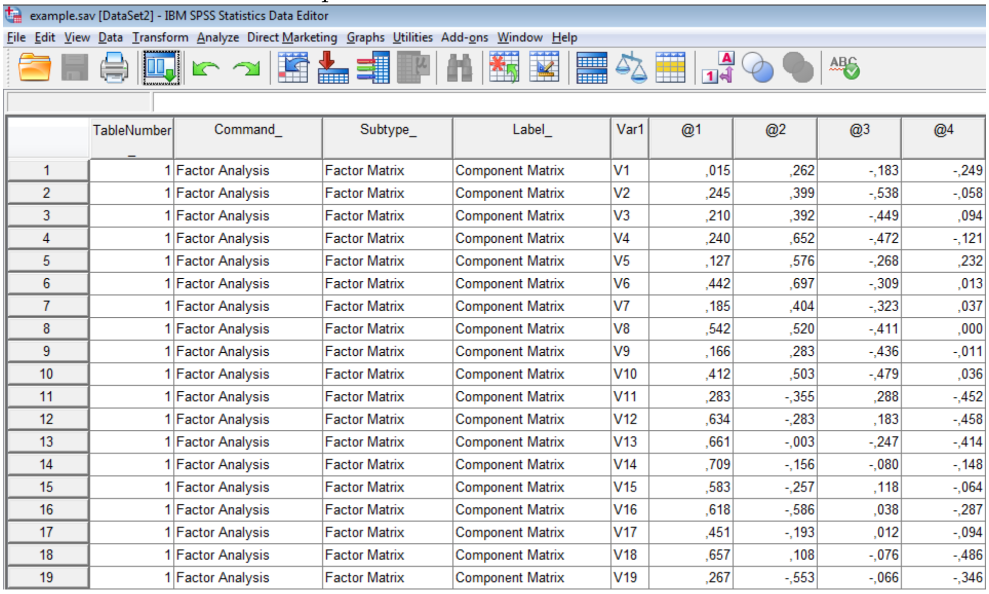

example.sav [DataSet2] - IBM SPSS Statistics Data Editor

	TableNumber_	Command_	Subtype_	Label_	Var1	@1	@2	@3	@4
1	1	Factor Analysis	Factor Matrix	Component Matrix	V1	,015	,262	-,183	-,249
2	1	Factor Analysis	Factor Matrix	Component Matrix	V2	,245	,399	-,538	-,058
3	1	Factor Analysis	Factor Matrix	Component Matrix	V3	,210	,392	-,449	,094
4	1	Factor Analysis	Factor Matrix	Component Matrix	V4	,240	,652	-,472	-,121
5	1	Factor Analysis	Factor Matrix	Component Matrix	V5	,127	,576	-,268	,232
6	1	Factor Analysis	Factor Matrix	Component Matrix	V6	,442	,697	-,309	,013
7	1	Factor Analysis	Factor Matrix	Component Matrix	V7	,185	,404	-,323	,037
8	1	Factor Analysis	Factor Matrix	Component Matrix	V8	,542	,520	-,411	,000
9	1	Factor Analysis	Factor Matrix	Component Matrix	V9	,166	,283	-,436	-,011
10	1	Factor Analysis	Factor Matrix	Component Matrix	V10	,412	,503	-,479	,036
11	1	Factor Analysis	Factor Matrix	Component Matrix	V11	,283	-,355	,288	-,452
12	1	Factor Analysis	Factor Matrix	Component Matrix	V12	,634	-,283	,183	-,458
13	1	Factor Analysis	Factor Matrix	Component Matrix	V13	,661	-,003	-,247	-,414
14	1	Factor Analysis	Factor Matrix	Component Matrix	V14	,709	-,156	-,080	-,148
15	1	Factor Analysis	Factor Matrix	Component Matrix	V15	,583	-,257	,118	-,064
16	1	Factor Analysis	Factor Matrix	Component Matrix	V16	,618	-,586	,038	-,287
17	1	Factor Analysis	Factor Matrix	Component Matrix	V17	,451	-,193	,012	-,094
18	1	Factor Analysis	Factor Matrix	Component Matrix	V18	,657	,108	-,076	-,486
19	1	Factor Analysis	Factor Matrix	Component Matrix	V19	,267	-,553	-,066	-,346

3.3 Syntax options

The options for the syntax are in `Run_GPA.sps`. This file can be adjusted to change the options used in the `GPA.sps file`. When the `Run_GPA.sps` file

is opened, the syntax looks like this:

```
BEGIN PROGRAM.
import spss
spss.Submit(r"INSERT FILE='{path using / }GPA.sps'.")

theDict['gpa_pool'](
source_file = "{path using / + filename of the source file}" ,
component_names = "{names of components}" ,
variable_names = "{variable with the original variable names}" ,
M = {number of imputed datasets} ,

)

END PROGRAM.
```

At least five arguments need to be specified to successfully carry out the procedure. These arguments are explained below.

3.3.1 Specifying the File Locations

Two file locations need to be specified in order for the syntax to be run. After the `INSERT FILE` command the location of the `GPA.sps` file needs to be specified. Suppose the `GPA.sps` file is located in the folder

```
C:\Example\
```

change

```
spss.Submit(r"INSERT FILE='{path using / }GPA.sps'.")
```

into

```
spss.Submit(r"INSERT FILE='C:/Example/GPA.sps'.")
```

Note that forward slashes are used in the example. SPSS may not be able to locate the file if backward slashes are used. After source file has been specified, the location of the file with the component loadings needs to be specified as well. This is the file that was created in section 3.2.2 using the `OMS` command. Suppose this file is called `ExamplePCA.sav` file and is located in the folder

```
C:\Example\.
```

the line:

```
source_file = "{path using / + filename of the source file}" ,
```

must then be changed into:

```
source_file = "C:/Example/ExamplePCA.sav" ,
```

3.3.2 Specifying the Names of the Components

To specify the names of the components, adjust the following line:

```
component_names = "{names of components}" ,
```

by replacing {`names of components`} with the names of the variables holding the components you want to use. Separate these names by commas. For example, in `ExamplePCA.sav` these variables are names 1, 2, 3, and 4. To use these names, the following line can be used:

```
component_names = "@1, @2, @3, @4" ,
```

3.3.3 Specifying the Number of Imputations

Suppose the number of imputations is 10. The line

```
M = {number of imputed datasets} ,
```

must then be changed to:

```
M = 10 ,
```

Figure 11: *Data File* **Example.sav**.

	TableNumber_	Command_	Subtype_	Label_	Var1
1	1	Factor Analysis	Factor Matrix	Component Matrix	V1
2	1	Factor Analysis	Factor Matrix	Component Matrix	V2
3	1	Factor Analysis	Factor Matrix	Component Matrix	V3
4	1	Factor Analysis	Factor Matrix	Component Matrix	V4
5	1	Factor Analysis	Factor Matrix	Component Matrix	V5
6	1	Factor Analysis	Factor Matrix	Component Matrix	V6
7	1	Factor Analysis	Factor Matrix	Component Matrix	V7
8	1	Factor Analysis	Factor Matrix	Component Matrix	V8
9	1	Factor Analysis	Factor Matrix	Component Matrix	V9
10	1	Factor Analysis	Factor Matrix	Component Matrix	V10
11	1	Factor Analysis	Factor Matrix	Component Matrix	V11
12	1	Factor Analysis	Factor Matrix	Component Matrix	V12
13	1	Factor Analysis	Factor Matrix	Component Matrix	V13

3.3.4 Specifying the Variable that Includes the Original Variable Names

After using the `OMS` option to create a file with the factor matrices, a variable will be created including the original variable names. In `ExamplePCA.sav` this variable is called `Var1` (see Figure 11). To specify that the original variable names are in `Var1`, change

```
variable_names = "{variable with the original variable names}" ,
```

into

```
variable_names = "Var1" ,
```

3.4 Optional Arguments

Besides the five required arguments there are 10 optional arguments. They differ from the mandatory arguments in that they have a default setting that will be used if nothing is specified.

3.4.1 Specifying the Rotation Method

This command supports two options: printing only the centroid solution of the Generalized Procrustes Analysis or printing the centroid solution followed

by a Varimax rotation as well. Use the following line to print the Varimax rotated centroid solution, add the following line:

```
rotation = "var" ,
```

to the syntax. Thus, suppose that we want to display the Varimax rotated solution of the `ExamplePCA.sav` file, the syntax must be modified into:

```
BEGIN PROGRAM.
import spss
spss.Submit(r"INSERT FILE='C:/Example/GPA.sps'.")

theDict['gpa_pool'](
source_file = "C:/Example/ExamplePCA.sav" ,
component_names = "@1, @2, @3, @4" ,
M = 5 ,
variable_names = "Var1" ,
rotation = "var" ,

)

END PROGRAM.
```

When the rotation argument is not added, only the unrotated pooled solution is provided. However, displaying only the unrotated solution can also be specified by adding `rotation = "no"` rather than adding `rotation = "var"`.

3.4.2 Specifying whether the Original Dataset is Included

When multiple imputation is used on a dataset with missing values in SPSS, SPSS adds the imputed datasets below the original dataset. The standard setting for the `GPA.sps` file is to ignore the first dataset for the Generalized Procrustes Analysis. However, if the first dataset is not the original dataset with missing data, the following line must be added to the syntax:

```
original_dataset = "no" ,
```

Thus, suppose that the `ExamplePCA.sav` did not include the original dataset, the complete syntax would look like:

```
BEGIN PROGRAM.
import spss
spss.Submit(r"INSERT FILE='C:/Example/GPA.sps'.")

theDict['gpa_pool'](
source_file = "C:/Example/ExamplePCA.sav" ,
component_names = "@1, @2, @3, @4" ,
M = 5 ,
variable_names = "Var1" ,
original_dataset = "no" ,

)

END PROGRAM.
```

If the first dataset in the file is the original dataset with missing values, this option can either be set to "yes" or removed altogether. In this case the syntax will revert to the default setting of "yes". Alternatively, the options "y" and "n" may also be used.

3.4.3 Saving the Combined Results in a Data File

The dataset with the results of the Generalized Procrustes Analysis is stored in a new SPSS data file. The default value of this parameter is "no", which does not save the SPSS data file. If this option is chosen, the file can still be saved manually. Suppose the desired file name is outcome.sav and the desired location is `C:\Example\`. To save the data file automatically, add the following line:

```
save_file = "C:/Example/outcome.sav" ,
```

to the syntax:

```
BEGIN PROGRAM.
import spss
spss.Submit(r"INSERT FILE='C:/Example/GPA.sps'.")

theDict['gpa_pool'](
source_file = "C:/Example/ExamplePCA.sav" ,
component_names = "@1, @2, @3, @4" ,
M = 5 ,
```

```
variable_names = "Var1" ,
save_file = "C:/Example/outcome.sav" ,

)
```

3.4.4 Graphical Options

The syntax has five graphical options. In the first two options it can be specified whether the results of either the Generalized Procrustes Analysis or the Generalized Procrustes Analysis with Varimax rotation are displayed in loading plots or not. For both options, the default value is "no". If the loading plots are shown, the default option is to display the centroids and convex hulls of the solutions for all the variables. To display the loading plots of the Generalized Procrustes Analysis, add the following line:

```
graph_gpa = "yes" ,
```

To display the loading plots of the Generalized Procrustes Analysis with Varimax rotation, add

```
graph_varimax = "yes" ,
```

If the loading plots are displayed, the default setting is to show both the centroids and the convex hulls. To disable displaying the convex hulls, use:

```
convex = "no" ,
```

To disable displaying the centroid solutions, use:

```
centroid = "no" ,
```

Alternatively, the options "y" and "n" may also be used. The last graphical option provides the possibility to show only a subset of the original variables. If the original dataset has many variables, the loading plots can become cluttered and difficult to interpret. With this option a subset of variables may be specified.For example, if you only want to see the variables `V1`, `V3`, and `V4`, add the following line:

```
subset = "V1, V3, V4" ,
```

Figure 12: *Example of a Graph Created by the Syntax.*

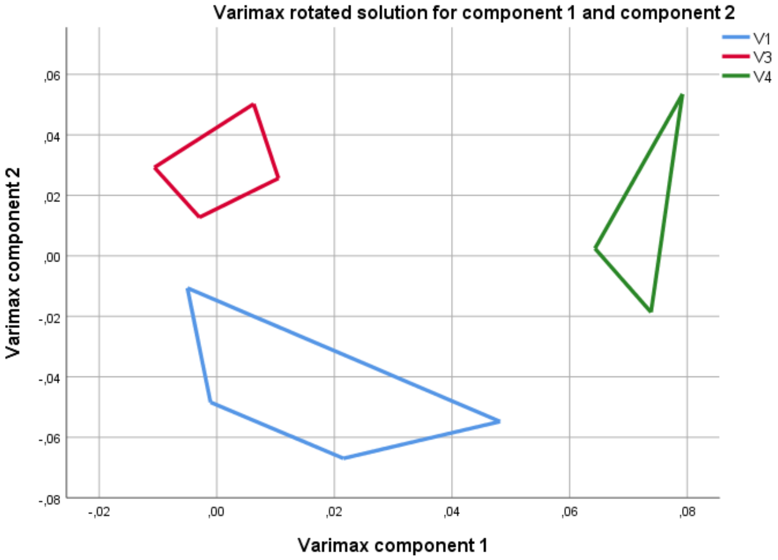

Using these settings the complete syntax looks like:

```
BEGIN PROGRAM.
import spss
spss.Submit(r"INSERT FILE='C:/Example/GPA.sps'.")

theDict['gpa_pool'](
source_file = "C:/Example/ExamplePCA.sav" ,
component_names = "@1, @2, @3, @4" ,
M = 5 ,
variable_names = "Var1" ,
graph_gpa = "n" ,
graph_varimax = "y" ,
convex = "y" ,
centroid = "no" ,
subset = "V1, V3, V4" ,

)
```

Using this syntax, the output for `Example.sav` looks as in Figure 12.

3.4.5 Specifying the Maximum Number of Iterations

Both the maximum number of iterations for the Generalized Procrustes Analysis algorithm and for the Varimax rotation algorithm in order to converge may be specified. The default maximum number of iterations for both procedures is 1000. The maximum number of iterations for the Generalized Procrustes Analysis algorithm can be changed. To set the maximum number of iterations of the GPA algorithm to, say, 3000, use:

```
max_iterations_gpa = 3000 ,
```

To set the maximum number of iterations of the Varimax algorithm to, say, 2000, use:

```
max_iterations_varimax = 2000 ,
```

Applied to dataset `Example.sav` the complete syntax looks like:

```
BEGIN PROGRAM.
import spss
spss.Submit(r"INSERT FILE='C:/Example/GPA.sps'.")

theDict['gpa_pool'](
source_file = "C:/Example/ExamplePCA.sav" ,
component_names = "@1, @2, @3, @4" ,
M = 5 ,
max_iterations_gpa = 3000 ,
max_iterations_varimax = 2000 ,

)
```

3.4.6 Specifying Several Optional Arguments Simultaneously

One may want to use several of the previously discussed optional arguments simultaneously. For example, print the varimax rotated solution, display both the unrotated and rotated solutions with their centroids and convex hulls for variables `V1`,`V3`, `V4` in graphs, and let the GPA and Varimax algorithms run for 3000 and 2000 iterations, respectively:

```
BEGIN PROGRAM.
import spss
spss.Submit(r"INSERT FILE='P:/imputation/GPA/GPA.sps'.")
```

```
theDict['gpa_pool'](
source_file = "P:/imputation/GPA/ExamplePCA.sav" ,
component_names = "@1, @2, @3, @4" ,
variable_names = "Var1" ,
M = 5 ,

rotation = "var" ,
graph_gpa = "y" ,
graph_varimax = "y" ,
convex = "y" ,
centroid = "y" ,
subset = "V1, V3, V4" ,
max_iterations_gpa = 3000 ,
max_iterations_varimax =  2000 ,

)

END PROGRAM.
```

References

Gower, J. C. (1975). Generalized Procrustes Analysis. *Psychometrika, 40,* 33–51.

Rubin, D. B. (1987). *Multiple imputation for nonresponse in surveys.* New York: Wiley.

Ten Berge, J. M. F. (1977). Orthogonal Procrustes rotation for two or more matrices. *Psychometrika, 42,* 267–276.

Van Ginkel, J. R. & Kroonenberg, P. M. (2014). Using Generalized Procrustes Analysis for Multiple Imputation in Principal Component Analysis. *Journal of Classification, 31,* 242–269.

Van Ginkel, J. R., Van der Ark, L. A., & Sijtsma, K. (2007). Multiple imputation for item scores when test data are factorially complex. *British Journal of Mathematical and Statistical Psychology, 60,* 315–337.
